# A Scoping Review of Facilitators of Multi-Professional Collaboration in Primary Care

**DOI:** 10.5334/ijic.3959

**Published:** 2018-08-30

**Authors:** Monica Sørensen, Una Stenberg, Lisa Garnweidner-Holme

**Affiliations:** 1 Department of Nursing and Health Promotion, Oslo Metropolitan University, St. Olavs Plass, 0130 Oslo, NO; 2 The Norwegian Directorate of Health, St. Olavs Plass, 0130 Oslo, NO; 3 The Norwegian National Advisory Unit on Learning and Mastery in Health, Oslo University Hospital, Trondheimsveien 235, 0586 Oslo, NO

**Keywords:** multi-professional collaboration, multi-professional communication, team-based care, primary care

## Abstract

**Introduction:**

Multi-professional collaboration (MPC) is essential for the delivery of effective and comprehensive care services. As in other European countries, primary care in Norway is challenged by altered patient values and the increased expectations of health administrations to participate in team-based care. This scoping review reports on the organisational, processual, relational and contextual facilitators of collaboration between general practitioners (GP) and other healthcare professionals (HCPs) in primary care.

**Methods:**

A systematic search in specialist and Scandinavian databases retrieved 707 citations. Following the inclusion criteria, nineteen studies were considered eligible and examined according to Arksey and O’Malley’s methodological framework for scoping reviews. The retrieved literature was analysed employing a content analysis approach. A group of stakeholders commented on study findings to enhance study validity.

**Results:**

Primary care research into MPC is immature and emerging in Norway. Our analysis showed that introducing common procedures for documentation and handling of patient data, knowledge sharing, and establishing local specialised multi-professional teams, facilitates MPC. The results indicate that advancements in work practices benefit from an initial system-level foundation with focus on local management and MPC leadership. Further, our results show that it is preferable to enhance collaborative skills before introducing new professional teams, roles and responsibilities. Investing in professional relations could build trust, respect and continuity. In this respect, sufficient time must be allocated during the working day for professionals to share reflections and engage in mutual learning.

**Conclusion:**

There is a paucity of research concerning the application and management of MPC in Norwegian primary care. The work practices and relations between professionals, primary care institutions and stakeholders on a macro level is inadequate. Health care is a complex system in which HCPs need managerial support to harvest the untapped benefits of MPC in primary care. As international research demonstrates, local managers must be supported with infrastructure on a macro level to understand the embedding of practice and look at what professionals actually do and how they work.

## Introduction

As the central pillar of healthcare systems worldwide, primary care provides entire populations with continuous, comprehensive and coordinated care services [1]. However, the development of new and more effective collaborative working arrangements is deemed necessary to serve imminent epidemiological and demographical demands [2]. It is envisioned that multi-professional team-based care approaches, in which professionals from different disciplines benefit from each other’s complementary skills and work towards common goals, will improve patient and provider satisfaction and the standards of care for persons with complex medical needs, such as mental illness, disabilities, multimorbidity or addictions [3,4,5,6]. Likewise, interdisciplinary teamwork is regarded as a core skill for future healthcare professionals (HCPs) beyond the command of knowledge and facts [7].

Globally, there is an increasing recognition that primary care and GPs should be organised in such a way as to assume full coordinating responsibility for entire populations of patients [1]. It is therefore crucial to supply primary care, and general practice in particular, with the necessary resources, technology and leadership, permitting the provision of coordinated and comprehensive care services.

Mobilising and transforming care services in accordance with altered values and increased expectations from patients as well as health administrations is a global challenge [8]. Because of inadequate care integration, leadership and multi-professional collaboration (MPC) in primary and secondary care, the needs of Norwegian patients for coordinated and integrated primary care services are not being sufficiently met [9,10,11,12]. One of the main challenges is the lack of collaborative procedures across institutions and the delayed implementation and adoption of technology [13]. For example, homecare nurses (HCN), accident & emergency departments (A&Es), pharmacies and general practices have different and separate electronic health records. To better understand what advancements are necessary to successfully improve MPC, we have explored literature that reports HCPs’ experience of professional collaboration involving GPs in Norwegian primary care. In particular, we have evaluated the relational, processual, organisational and contextual dimensions of professional collaboration, inspired by Reeves et al’s framework for inter-professional collaboration [14]. Previous application of this framework has been used, for example, in studies describing the perspectives of GPs of their role in the primary care team, the factors that facilitate and hinder teamwork [15] and in examining the perspectives and experiences of family health team members regarding inter-professional collaboration and perceived benefits [16].

When studying the healthcare system, understanding system-level coordination and the relational and functional aspects of the system is fundamental. Reeves et al’s framework describes the organisational, relational, contextual and processual domains of professional collaboration. The **organisational** aspect pertains to factors affecting the local organisational environment in which professionals work. The **relational** domain is linked to how factors such as power, hierarchy, socialisation, leadership and participation in collaborative practices are understood. The **contextual** level depends upon national, regional, political or professional authorities and the priorities they have that foster collaboration, such as policy papers, strategies, funding or support of local multi-professional activities. **Processual** aspects of collaboration are those pertaining to time, space, proximity, task complexity and how this affects teamwork.

To our knowledge, this is the first scoping review to explore the facilitators of MPC between GPs and other HCPs in primary care in a Nordic country. We acknowledge that there are many international publications that target the facilitators and barriers of multi-professional care. Nonetheless, implementation of multi-professional teamwork and education remains a global challenge [17]. This study brings important perspectives on critical organisational, relational, contextual and processual domains of MPC from a healthcare system in which multi-professional collaboration is emerging.

### Aim and review questions

The purpose of this scoping review is threefold: First, we will fill in the gaps of knowledge regarding which professionals are involved in MPC with Norwegian GPs and explore their collaborative procedures. Second, we will identify the organisational, processual, relational and contextual facilitators which promote the collaboration of GPs with other HCPs in Norwegian primary care as experienced by the involved professionals. Third, the comments of national stakeholders on the findings and comparison of the results with international literature will be performed to demonstrate potential policy implications for improving collaborative practice in primary care.

Our research questions were as follows:

- What are the characteristics (study design, methodology and participating HCPs) of studies involving the participation of GPs in MPC in Norwegian primary care?
- From the perspective of HCPs working in Norwegian primary care, what are the main organisational, processual, relational and contextual facilitators pertaining to MPC involving GPs?

## Methods

### Design and settings

We followed Arksey & O’Malley’s and Colquhoun et al’s frameworks for performing scoping reviews [18,19]. Scoping reviews are useful for mapping the main sources and key concepts of heterogenic or emerging fields of research and to demonstrate research areas in which there are a dearth of evidence for policy makers, practitioners and consumers. The framework suggested by Arksey & O’Malley offers five stages in which to carry out a scoping review. Stage 1: Identifying the research question. Stage 2: Identifying relevant studies. Stage 3: Study selection. Stage 4: Charting the data. Stage 5: Collating, summarising and reporting the results. An additional sixth optional element is that of consultation with practitioners and consumers. We sought publications involving GPs in MPC in primary care, published in international, Scandinavian and Norwegian professional journals, as well as municipal and governmental reports. Quality appraisal does not typically restrict the inclusion of studies in scoping reviews [19]. The aim of this review was to identify the range and subject matter of literature in the topic of interest. Thus, study quality, design or methodology did not affect study inclusion.

Norwegian health and social care provides universally-accessible public services in accordance with the Scandinavian Welfare Model and has one of the highest densities of physicians and nurses in Europe [20,21]. Norwegian municipalities enter into contracts with individual, self-employed GPs, who receive a combination of capitation (~35% of income), fee-for-service (~35%), and out-of-pocket payments from patients (~30%) [22]. The average general practice has 3.6 GPs, 0.8 medical secretaries per GP and an average of 1,130 patients [23,24]. Compared to Finland and Sweden, where specialist nurses have extensive responsibilities for the care of persons suffering from common chronic diseases such as diabetes, asthma and chronic obstructive pulmonary disease (COPD), nurses in Denmark and Norway are not traditionally delegated independent tasks in general practice. This may be due to the professional culture and to a lack of reimbursement for nursing services in general practice [10].

### Identifying relevant studies

We performed several pilot searches to improve the final search as outlined by Colquhoun et al. (keywords and databases can be found in Table 1) [19]. Adjustments involved removing specifications (diagnoses) from target groups concerning multi-professional follow-up, specifying the professionals’ background and adjusting the publication time limit. These refinements were developed in consultation with an experienced medical librarian.

**Table 1:** Keywords and research databases used in systematic searches.

Keywords	Databases (from 2000 – week 3 to July 2017)

“Family Physician”	MEDLINE (OVID)
“General Practice”	CINAHL (OVID)
“Primary Care”	EMBASE
“Dietitians”	Epistemonikos
“Laboratory staff”	PSYCHINFO
“Medical laboratory personnel”	Web of Science
“Medical secretaries”	
“Nurses”	
“Nutritionists”	
“Occupational therapists”	
“Physical therapists”	
“Social workers”	
“Pharmacists”	

The search strategy may be found in Appendix 1. The Scandinavian databases SveMed and NorArt were manually searched using the same terms and criteria. Manual searches were also performed in the websites of Norwegian governmental bodies and municipalities. In addition, reference lists of the articles screened in full text were reviewed.

### Study selection

Articles in English, Norwegian, Swedish or Danish describing MPC in primary care in Norway published after 2000 until July 2017 were sought. For the purpose of this selection, MPC was defined as any cooperation between two or more professionals involving patient care or quality improvement of patient care. Abstracts obtained from the initial searches were independently reviewed by two investigators (US and MS). During the abstract review process, the merits and significance of articles for which there was disagreement were discussed until both investigators agreed. Reference lists of reviewed articles were manually examined for further studies by MS. Articles that reported from the perspective of secondary care or that lacked GP involvement were immediately excluded, as well as single abstracts, comments, study protocols and posters.

### Charting the data

Full-text articles were reviewed by two independent investigators who met regularly to discuss study inclusion (US and MS). A data extraction form was developed by the same two investigators based on experience and by using the included studies to continuously adjust the form. The form facilitated the comparison and analysis of data from the chosen articles and was revised throughout the reporting process to improve accuracy and specificity of the analysis. Data extraction and coding were mainly performed by MS over several rounds and reviewed by US (see Appendix 2 for the full data extraction form). Numerous discussions were held between investigators during the extraction stage to confirm the consistency of the extracted information with the aim of the study. All extracted information was registered and compiled in an electronic spreadsheet. We followed the suggestion by Colquhoun et al. of collating and presenting data in three stages: 1. Descriptive numerical summary analysis and qualitative content analysis. 2. Reporting of results referring to the research questions. 3. Interpreting the implications of the findings for future research, practice and policy [19].

### Collating, summarising and reporting the results

This is a mixed methods–mixed research scoping review in that it reports findings from qualitative, quantitative and mixed methods studies and the mode of synthesis uses qualitative and quantitative approaches to integrate these findings [25]. A content analysis guided the data interpretation, which focused on the main organisational, processual, relational and contextual facilitators of MPC as described in the retrieved publications [26]. Colquhoun et al. recommend that consulting stakeholders is an essential step before disseminating the results of scoping reviews. The purpose of the consultations was to broaden our understanding of the results and improve study validity. Stakeholder names, titles and positions are given in Appendix 3.

### Literature selection overview

**Figure 1:**
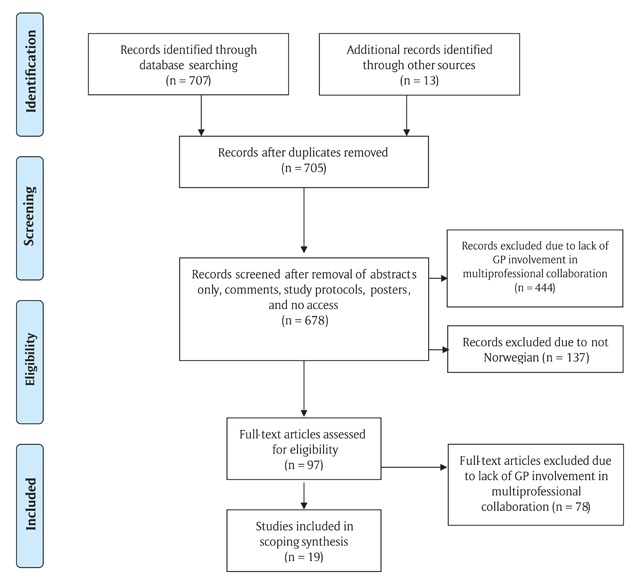
Search flow for multiprofessional collaboration in primary care involving general practitioners experiences.

In total, 707 titles or abstracts were identified. Full-text papers of 83 articles were retrieved from the main searches for detailed evaluation. Another twelve citations were detected by examining reference lists. Two studies were identified through manual searches in governmental websites [27,28]. Thus, 97 full-text articles were screened before two investigators individually agreed that 19 studies, published between 2000 and 2017, met the eligibility criteria. Figure 1 provides a Prisma flowchart of the literature selection process.

## Results

This scoping review sought to explore HCPs’ experience of MPC involving GPs in Norwegian primary care. In particular, the review examined professionals’ perceptions of the organisational, processual, relational and contextual facilitators of collaboration that will help advice policy development and a successful implementation of MPC.

### Descriptive summary of study characteristics and involved participants

None of the included studies involved a team-based care intervention within general practice. Hence, studies either involved GPs collaborating with other HCPs outside general practice or they reported from non-interventional collaboration in general practice, e.g. initiatives of quality development. The number of participants in the studies retrieved varied from 7 [29] to 1,633 [30]. Eleven studies involved an intervention, the remaining eight aimed to describe HCP’s experience of MPC. Twelve studies were published in Norwegian only.

Six studies reported both qualitative and quantitative data [31,32,33,34,35,36], ten qualitative only [27,29,37,38,39,40,41,42,43,44], and three quantitative only [28,30,45]. The numbers and characteristics of the participants involved in the included publications are given in Table 2.

**Table 2:** Study design and distribution of participants.

	Mixed methods* (N = 6)	Qualitative (N = 10)	Quantitative (N = 3)	Total (N = 19)

**GPs/physicians**	334	61	1,663	2,058
**Nurses**^§^	1,017	68	789	1,874
**CPWs**	519			519
**Physiotherapists**		28		28
**Secretaries/lab. assistants**		21		21
**Patients**	474	179	554	1,207

*GP: General practitioner; CPW: Child protection worker*. * One study did not report the distribution of responders among GPs, HCNs and municipal case managers (n = 32) (35). ^§^ Includes HCNs, managers in homecare services and cancer coordinators.

All the mixed methods studies used questionnaires on data collection. Two studies performed focus-group or individual interviews in conjunction with the questionnaire. Studies following a qualitative design used a variety of data collection methods: focus groups, descriptive exploratory design, questionnaires, meeting reports, comments from patients’ medical records, narratives, telephone interviews, observations and semi-structured interviews. The three quantitative studies followed a questionnaire-based, cross-sectional group comparison design.

Appendix 4 presents the aims, design and study conclusions of the included interventions and surveys.

### Facilitators for multi-professional collaboration in primary care

This review retrieved a heterogenic collection of literature illustrating team-based care as an emerging and understudied field of research. The identified studies depict important organisational, processual, relational and contextual facilitators applicable to the emerging field of multi-professional education and collaboration. The findings are summarised in Table 3.

**Table 3:** Organisational, processual, relational and contextual facilitators of MPC in primary care.


**Organisational facilitators of multi-professional collaboration** Establish **procedures** for inter-professional meetings and documentation and handling of patient data (e.g. e-communication) Facilitate **knowledge sharing** between disconnected professionals Establish local, specialised **multi-professional teams** Establish system-level foundation that **supports local management and leadership** of MPC

**Processual facilitators of multi-professional collaboration** Enhance **collaborative skills** before introducing new professional teams, roles and responsibilities Develop **common quality-management systems** across institutions Allocate sufficient **time** for professionals to share reflections and engage in mutual learning

**Relational and contextual facilitators of multi-professional collaboration** Invest in **professional relations** that build trust, respect and continuity Improve professionals’ **knowledge of each other’s skills and roles** through inter-professional education **Educate patients** about the benefits of MPC


#### Organisational facilitators of multi-professional collaboration

Organisational capabilities and structures describe dynamic elements in the local environment subject to the success of integrated care delivery and the support of MPC [46,47].

Collaborative practice is effective when there are opportunities for shared decision-making and routine meetings [48]. We found that professionals working in different primary care institutions lacked a shared *modus operandi* for documentation and handling of patient data, shared decision making [27,29,31,33,35] and e-communication [44,45]. One study suggested that secondary care should develop protocols and individual patient care plans at discharge, which may facilitate uniform cancer rehabilitation in municipal health and social services [31]. Two studies addressed e-messaging between HCNs and GPs [44,45]. Results from the study of Borgen et al. indicated that e-messages increased the frequency, quality and inter-professional interaction between GPs and nurses [44].

The results showed that joining currently disconnected professionals, extending their professional responsibilities and facilitation of knowledge sharing are untapped resources in primary care that could increase the level of work satisfaction of professionals [27,40], quality of care [28,32,42,43] and improve preventive care planning [32,38]. However, the habitual way in which professionals operate must be synchronized and their modes of communication systemised. For example, in Magnussen’s study, GPs were concerned about interrupted communication of patient care information following the introduction of point-of-contact-nurses, which potentially posed a threat to patient safety [27].

Most studies in this review were locally initiated without system-level foundation. Several studies reported that inadequate leadership inhibited new methods of MPC implementation [48,49]. There was a consistent lack of system- and policy-level support for integrating the projects with the overall municipal health and social care system. For example, the implementation of cancer coordinators and their services was not sufficiently publicised by local authorities and the HCPs had to dedicate time to implementing and notifying other primary care professionals about their services [36]. Quality improvement projects in general practice were terminated due to lack of municipal leadership [39] and a well-functioning team-based model for diabetes care was not shared and scaled up [41].

#### Processual facilitators of multi-professional collaboration

Processual aspects of collaboration pertain to situational factors such as time, proximity and task complexity. Several studies sought to enhance the professional skills of physicians and non-physician professionals by introducing new work alliances and responsibilities which paved the way for shared learning either within institutions, e.g. nurses commencing diabetes controls in general practice [41] or across institutions, e.g. pharmacists participating in medical reviews in case conferences with GPs and nurses [43]. The success of introducing new skill mixes depends on the collaborative skills of all members of the team [48]. Another critical determinant for succeeding in improving MPC relates to the professionals’ time [49]. In several studies, HCPs engaged in quality development after their working hours or reported that finding time during the day was an obstacle to participating in sharing reflections and learning from collaborative partners [35,38,39,43].

Extending the roles of professionals may improve the quality of care. One study reported the advantages of introducing new working practices to improve quality improvement projects in general practice [40]. By engaging all staff members, the practice managed to reduce the number of errors considerably and improve the practice’s collaboration with the HCSs. Another example of quality improvement was the establishment of local multi-professional dementia teams which increased the number of dementia diagnoses [32], whereas Syse & Moshina’s study showed that extending nurses responsibilities may improve municipal cancer rehabilitation [36]. The study by Bell et al. showed that nurses and GPs were unaware of the benefits of engaging pharmacists in reviewing the pharmacological therapy of patients with multimorbidity and polypharmacy [43].

The collaboration between HCNs and GPs was reported as being challenging in several studies [27,28,35,42,44]. Nurses in particular reported that contacting GPs was difficult and time consuming [42,44]. However, with processual changes and new routines, the collaboration was improved [27,28]. For example, entrusting specific HCNs to act as a point of contact with the GPs on behalf of all the other nurses reduced the number of phone calls between HCS and general practice, increased collaborative efficiency and reduced the amount of unnecessary medication for patients receiving HCS [28]. In Kvamme & Lothe’s study, a shared quality-management system covering local care procedures in HCS and general practice was developed to improve communication and coherency in clinical procedures [35].

#### Relational and contextual facilitators of multi-professional collaboration

Relational and contextual facilitators were found to be closely connected. New skill mixes and expansion of the professional’s roles requires a cultural transformation of GPs’ approach to other professionals. From being used to working and accommodating most patients’ needs independently, GPs must acknowledge that collaboration with non-physician professionals may offer patients more targeted health care [31,32,41]. As an example, two studies highlighting the collaboration between GPs and physiotherapists reported poor levels of communication, knowledge and collaborative working arrangements between these professions [29,33]. Both groups of professionals reported confusing jargon and use of terminology. The physiotherapists complained about the GPs’ inadequate description of patients’ symptoms and that diagnostic codes were of little use when referring patients with vague and complex complaints to a physiotherapist. The authors called for a discontinuation of the hierarchical and power-related dynamics between the two groups of professionals and the establishment of new collaborative procedures for referrals from GPs and reports from physiotherapists to increase the level of satisfaction and the perception of the usefulness of collaborating with each other. In this regard, investing in the professional’s partnerships and knowledge of each other’s skills and roles is a relational as well as a processual facilitator for collaboration.

While time was found to be an important processual facilitator for MPC, important relational dimensions were trust, respect, collaborative skills (e.g. focusing on sharing knowledge, being open to others influencing your decisions, appreciating each other’s efforts and trusting each other’s skills) and physical proximity [29,34,40]. Interestingly, we found that more extensive experience of practice enhanced the non-physician’s perception of the quality of collaboration and communication with GPs [36]. Similarly, GPs satisfaction with the collaboration with nursing homes positively correlated with relational continuity [30]. The implementation of new interventions was also found to be easier in municipalities in which the HCPs were already well known among the target group [38]. This could indicate a culture of scepticism and a lack of curiosity and openness among different professional disciplines. For example, in the study by Magnussen, in which nurses and GPs had monthly meetings about roles and responsibility, nurses experienced an increased endorsement of their professional work and an increased willingness by GPs to discuss a broader range of topics, compared to their regular interaction with GPs [27]. Thus, investing in inter-professional relationships may have positive effects on the level of professional work satisfaction.

## Discussion

This comprehensive scoping review revealed a shortfall in published research about the collaboration of GPs with other HCPs in primary care. The included studies provide some generalisable facilitators from a range of examples of MPC in primary care. The spectrum of initiatives reflects a healthcare system progressing towards integrated care delivery and its readiness for change and organisational maturity, which are different from systems that represent integrated care sustainment. The advancement of integrated care requires an understanding of the underlying adaptive organisational, processual, contextual and relational capabilities that support collaboration at the micro (patient, provider), meso (organisational/institutional) and macro (system/policy) level [50] The limited number of identified publications clearly highlights the need for further exploration of this area of the Norwegian healthcare system.

### Improving collaborative practices requires system-level infrastructure

Developing new organisational infrastructure is crucial in integrated care delivery [47,51]. It has been proposed that creating collaborative and integrated care involves an *a priori* structuring for flexibility, meaning that healthcare systems are made more sustainable when they are ready for continuous transitions into even more complex services [52].

Workforce planning, inter-professional education and responsive monitoring of quality improvement through audits and feedback are organisational domains that have been proven critical in improving the dynamics in local healthcare services [52,53]. We found that the work practices of HCPs did not accommodate collaboration or teamwork but were constrained by lack of time and diverging modes of professional practice among pillarised institutions. In Norway, inter-professional education is only at the experimental stage with no legislative support [54]. Nor is there any audit and feedback programme that monitors the quality of primary care services.

The studies in this review have illustrated that exploring new professional constellations and work practices could improve care efficiency and the level of work satisfaction of professionals [28,38,40,43]. However, this will amplify the demand for the establishment of collaborative procedures and the necessary infrastructure to facilitate effective communication and professionals’ access to up-dated patient data. The HCPs informing this review were generally left alone with the responsibility for planning and implementing initiatives to improve collaboration and quality of care, which may demonstrate ineffectual management at a system-level and unsatisfactory local coordination and leadership of the collaborative efforts of HCPs. As others have shown, initiatives in the healthcare setting continue to be developed in isolation rather than interactively at micro- and macro-levels. This is an inefficient and expensive undertaking that rarely translates into higher quality of care [55].

According to our analysis, HCPs were aware of the collaborative and communicative shortcomings among professionals in primary care. It was shown that although GPs reported unsatisfactory collaboration with nursing homes [30,37,42], HCNs [27,31,37,45] and physiotherapists [29], they lacked the time, experience and training to engage in improving their practice [30,31]. Attending to the intrinsic capacities, barriers, needs and interdependencies of primary care requires a systemic approach in which local health and social care managers share responsibility with professionals and lay people in boosting clinical outcomes. Indeed, leadership is essential to encourage the use and implementation of innovative workflows, collaborative structures and to support long-term quality improvement [56].

In this regard, experience from the UK may prove valuable. In its efforts to improve integrated care, the NHS identified separated budgets, institutional organisation, professional separation, different cultures and lack of integrated data and information systems as the most significant barriers [57]. Taking these lessons into account and moving forward, the NHS has established a Leadership Centre that has coordinated and facilitated clinical leadership development programmes, clinical audits, risk management, user involvement, reflective practice and team reviews since 2001 [58,59]. Recently, local service managers from the NHS and the provider side have developed plans with the aim of transforming health and care in the communities they serve [60]. The initiatives include increasing the number of clinical pharmacists, physician associates and general practice nurses, as well as linking GPs with mental health therapists and expanding the number of practices working together in primary care networks. The commissioners’ mandate includes a five-year budget and evaluation plan in which the budget allocation is entrusted to the NHS commissioners directly to prevent political interference regarding the way in which funding is distributed [61]. Leadership by trained health service managers is regarded as a pivotal element in managing and running the commissioning and delivery of local health care [62].

### Successful MPC calls for formalised procedures of communication and collaboration

The processual aspects of collaboration are linked to how the actual working practices affect teamwork. Several of the initiatives included in this review focused on improving the processual efficiency of care, i.e. promoting more effectively the communication between GPs and HCNs [28,40] or improving collaboration between GPs and multi-professional teams for patients with complex needs [32,35,36,38,43]. Reallocation of tasks or new cooperative alignments between GPs and pharmacists [43], the HCSs [35], nurses at nursing homes [43] or the introduction of new responsibilities for other professionals in general practice [41], were found to be plausible in alleviating some of the pressure on GPs and to have synergetic effects on care procedures.

We found several important processual factors that affected MPC such as time, co-location and system complexity. Though time is a limitation in health care, the communicative procedures between GPs and other healthcare professionals did not generally support efficient collaboration [27,28,29,30,31,33,35,36,37,40,42,44,45]. However, formalising the procedures for collaboration reduced the amount of time HCNs and GPs spent on attempting to make contact with each other [27,40] and improved information exchange and care efficiency between municipal teams and GPs [27,28,32,38,40,43,44]. Moreover, the introduction of electronic messages could improve communicative efficiency if the collaborating partners can reach an agreement on how it should be used procedurally [44,45].

Co-location of professionals may facilitate improved care for frequent users and patients with complex needs [63,64]. The study by Graue described HCNs who received diverging advice from different physicians located in general practices and hospitals that left nurses feeling unsupported in the clinical setting. This may indicate that separation of professionals is a hinder to sharing standards of care and may impede nurses’ perception of professional support [37].

Health care is a complex system [65]. The components of complex systems interact nonlinearly over multiple scales and produce unexpected results. Hence, siloed programmes for managing health care will often fail [66]. The abandonment of the notion of nonlinearity, in which nonlinearity means that the output is greater than the sum of its parts, became evident in our analysis in the form of the neglect of patient transitions and communication channels, e.g. between psychomotoric physiotherapists [29] and chiropractors [33] and GPs, or between GPs and multi-professional organisations such as nursing homes [30]. However, what emerged from our findings is that differentiating facilitators and barriers for MPC between the functionality of the healthcare system, the capacity of working practices and the intervention itself, was a challenge.

### Facilitation of personal relationships requires a systemic approach

The relational domain is linked to how relations, leadership and hierarchy impact inter-professional collaboration. Indeed, the professional culture and people’s values and involvement were found to be associated with the success and failure of the included interventions. For example, oncology nurses in the analysed study by Syse & Moshina used a considerable amount of time on building networks and informing other healthcare institutions about their services [36]. It is surprising that the municipalities regard this as a beneficial and efficient application of nurses’ time and that inter-professional education and training activities are not put into practice.

To build trusting relationships and identify problem areas and inherent capabilities for collaboration, professionals must spend time together [67]. We found several examples indicating the importance of investing time in building professional relationships. One innovative example is the GP who underwent psychomotoric physiotherapy sessions to improve his knowledge about what patients would most benefit from in this therapeutic approach [29]. His commitment also improved the communication between the two professions. In studies in which municipal teams were established, professionals were given more time to discuss their clinical experiences. This contributed to personal growth, a greater sense of acknowledgement from other HCPs, and enhanced self-awareness [27,28,38,40,41,43]. Contrarily, a lack of attention to the principles of teamwork, such as shared values and goals, may lead professionals to invalidate the mode of practice of other professionals, as the study by Engedal et al. showed, in which GPs considered the practice of dementia teams screening for dementia as unnecessary and of little use to the patient [32].

Contextual dimensions relate to authorial support of local multi-professional activities. The potential of an organisation’s structural and tangible resources depend on intangible features such as individuals’ collective attitudes and relationships [68]. Though leadership has been suggested as an influential indirect factor in shaping the organisational environment and culture [60,69], research has shown that the established institutional structures and norms in health care render leadership problematic [70]. To overcome the policy imperatives, professional divisions and bureaucratic structures that interfere with the frontline managers’ ability to lead across boundaries and up hierarchies, organisational structures must be altered. This will not be achieved through piecemeal changes to job titles and responsibilities in isolation from the context in which these are to be enacted and calls for system-level management.

In confirming our findings, there seems to be a growing consensus that the successful implementation of initiatives that promote professional collaboration takes into account local contexts and the broader social, political, economic and cultural environment [50]. This entails the acquirement of overall knowledge about the needs of local communities and the existing barriers and facilitators of MPC. For example, several of the included studies reported that commissioning GPs in collaborative activities was challenging [35,38,43]. This is not to be understood to mean that physicians are not cooperating but is an invitation to further research the inhibiting and promoting mechanisms of how MPC may be more effective and how GPs can be more involved in the advancement of primary care.

### Summary of stakeholders’ comments

Four stakeholders representing governmental and municipal authorities and academia were asked to assess the results to increase study validity and broaden our understanding of today’s challenges regarding MPC in primary care (please see Appendix 3 for a presentation of the stakeholders’ backgrounds).

Two of the stakeholders emphasised the need for formalised structures and leadership in creating integrated municipal healthcare services and two stakeholders argued that the lack of experience, skills and resources in municipalities in taking charge of research projects is an obstacle to implementing new collaborative practices. They also commented that conditional terms, legislation and resource priorities are hindering development and innovation in municipal health care.

One stakeholder commented that primary care lacks common guidelines, modes of collaboration, IT systems and binding agreements that increase GP participation in multi-professional and municipal collaboration. It was noted that managing and developing integrated multi-professional primary care services is a municipal responsibility. One stakeholder stated that municipalities lack formal control of GPs, contrasting, for example, home-based care services in which municipalities coordinate and manage altogether.

### Implications of the results on policy and practice

Norway is striving to become a leader in the prevention and management of chronic, non-communicable diseases [71]. Although Norwegian citizens enjoy one of the highest per capita health expenditures in the world [20], only around 6% of the total current health expenditure is used on primary care. This is half of the OECD average of 12% and insufficient resource allocation poses a threat to the sustainability of our primary care services [23].

The integration of bottom-up and top-down governance in healthcare settings may help to overcome dysfunctionalities associated with efficiency and coordination of care [72,73]. Measures that enhance cooperation between national and local authorities in ways that improve the capability of municipalities to establish supportive relationships with HCPs is necessary to contain costs, improve the quality of care and offer more population-suitable care [74,75]. For example, engaging in the implementation of common procedures and legislation for MPC is a managerial role, commencing with an evaluation of the quality of care services and establishing remuneration plans that support teamwork, local quality improvement and the inter-professional sharing of knowledge [67]. Next, it is important to improve knowledge about the level at which organisational management should be placed and how managers should become involved in centralised or distributed decision-making.

The gap between what we know facilitates MPC and integrated care, compared to everyday practice, remains a major challenge for health systems [76]. Thus, implementation research emphasises the need to balance internal (end users) and external validity and to understand the interplay between science, HCP behaviour, the population under care and the local delivery environment in the adoption of new knowledge. This process requires extensive consultation, flexibility and front-end review beginning with a dialogue about needs and the cognitive apprehension on the relationship of HCPs with other HCPs, their attitudes, beliefs and motivation to collaborate [77].

It was remarkable how profession-oriented the included studies were, a point about which several stakeholders commented. A key challenge for governance constitutes its detachment from the realities surrounding professional–patient relationships and patient preferences [78]. Shifting from a volume-driven system to a system that achieves outcomes that matter to patients requires the impact on policy development of patient-reported outcomes and needs [79]. Governmental strategies [71,80,81], reforms [82] and legislation [83,84] generally include a high volume of ultimate goals and expectations regarding the development of integrated and person-centred care services. However, scant attention is usually paid to guidance in the processes of delivering such services [85] and the necessary underlying organisational capabilities and conditions [51]. For example, in Norway there is no national policy that supports health organisations in the management of inter-professional relationships or in inter-professional education. In this sense, we suggest applying the existing knowledge from the numerous evidence-based frameworks that have been developed to diagnose the level of maturity of healthcare systems and to guide actions of improvement for inter-professional collaboration [86,87,88], integrated care [89,90] and person-centred care [91].

### Possible pitfalls when reorienting professional relationships in health care

The reinforcement of collaborative practice in healthcare and institutional settings must be multi-faceted and take into account that the system is more than the sum of its parts [92]. Ignorance of this critical point relates to the lack of high-quality intervention studies which demonstrate that inter-professional work activities can have a meaningful impact on health outcomes [93]. Collaboration does not equate to increased specialisation or delegation of tasks, which may incur communicative or professional challenges, such as role blurring or power struggles [94]. As experience from the UK has shown, solely directing focus on active management or governmental incentives without engaging professionals in taking ownership of the necessary changes of practice, may be disadvantageous [95]. For example, the redistribution of roles alters professional identity and may reorient health care towards biomedical problems and the sets of tasks that must be accomplished to fulfil a set of quality indicators, and away from discourses that focus on the social character of general practice and the notion of a patient-centred approach [96].

The acceptance of health care as a complex adaptive system based on culturally, ethically, politically and economically-sensitive relationships, in which the relationships between parts of the system are regarded as being more important than the parts themselves, may be a key factor to successful implementation [92,97,98].

### Study strengths and limitations

The strengths of this review encompass a broad and thorough search strategy resulting from several initial searches and performed by a medical librarian. We argue that the scoping review methodology was well suited to answering our research questions and providing a knowledge synthesis that addresses the key concepts, types of evidence and research gaps related to this explorative field of research. Additionally, acknowledgements and comments from several relevant stakeholders improved the study validity.

This review has several limitations. We may not have identified all relevant publications despite our efforts to be as comprehensive as possible. There is an ongoing debate regarding the typology of inter-professional activity, as no common terminology or definitions exist [17]. Reeves et al. defines collaboration as a looser form of inter-professional work than, for example, teamwork, which requires a “shared accountability between individuals, some interdependence between individuals and clarity of roles and goals” [99]. Xyrichis et al. recently published a validation paper that sought to clarify the concept of inter-professional working in health care. They suggest new sub-categories of inter-professional work activities such as “collaborative partnership, “coordinated collaboration”, “delegative coordination” and “consultative coordination” [17]. We acknowledge that several of the interventions or work practices reported in this review do not completely comply with Reeves et al’s definition of collaboration and that the collaborative characteristics may satisfy the new sub-categories. However, as our search retrieved only a limited number of publications that fulfilled the inclusion criteria, we found it most appropriate to classify everything as “multi-professional collaboration”.

The classification of collaboration in the various articles may reflect different degrees of collaboration and other investigators may have included a slightly different set of articles than those included in the present review. Furthermore, the review topic is an emerging field in Norwegian primary care and most initiatives were dependent on local actors with short-term financial support and limited research skills. Hence, relevant project results may be left unpublished.

Conclusions made by original authors regarding the included studies were not subject to our scrutiny and others may interpret their findings differently. We adopted Arksey and O’Malley’s definition of scoping reviews and did not evaluate the qualitative or financial implications of the included study results. Readers should note that, typically, the number of participants in the included studies was low.

## Conclusion

While accounts of MPC are increasingly being reported in literature, this review identified only 19 studies that discuss the application and management of MPC in Norway. The analysis indicates that the relations between micro-level professionals, primary care institutions and macro-level stakeholders are inadequate and further national research is required to understand these processes. Health care is a complex system in which HCPs need managerial support to harvest the untapped benefits of MPC in primary care. As international research demonstrates, local managers must be supported in understanding the embedding of practice and looking at what professionals actually do, how they work and the preferences of patients in serving as facilitators in collaborative practices and healthcare development.

## Additional Files

The additional files for this article can be found as follows:

10.5334/ijic.3959.s1 Appendix 1 Search strategy.

10.5334/ijic.3959.s2 Appendix 2 Data extraction form.

10.5334/ijic.3959.s3 Appendix 3 List of consulted stakeholders.

## References

[1] Primary Health Care – Now. More Than Ever; 2008 Almaty, Kazakhstan: World Health Organization.

[2] Institute of Medicine Committee on Quality of Health Care in A. Crossing the Quality Chasm: A New Health System for the 21st Century, 2001; Washington (DC): National Academies Press (US). Copyright 2001 by the National Academy of Sciences. All rights reserved.

[3] Hopman, P, de Bruin, SR, Forjaz, MJ, Rodriguez-Blazquez, C, Tonnara, G, Lemmens, LC, et al. Effectiveness of comprehensive care programs for patients with multiple chronic conditions or frailty: A systematic literature review. Health Policy, 2016; 120(7): 818–32. DOI: 10.1016/j.healthpol.2016.04.002 27114104

[4] Metzelthin, SF, van Rossum, E, de Witte, LP, Ambergen, AW, Hobma, SO, Sipers, W, et al. Effectiveness of interdisciplinary primary care approach to reduce disability in community dwelling frail older people: cluster randomised controlled trial. BMJ: British Medical Journal, 2013; 347. 10.1136/bmj.f5264 PMC3769159 24022033

[5] Harris, MF, Advocat, J, Crabtree, BF, Levesque, JF, Miller, WL, Gunn, JM, et al. Interprofessional teamwork innovations for primary health care practices and practitioners: evidence from a comparison of reform in three countries. Journal of multidisciplinary healthcare, 2016; 9: 35–46. DOI: 10.2147/JMDH.S97371 26889085 PMC4743635

[6] Wagner, EH, Glasgow, RE, Davis, C, Bonomi, AE, Provost, L, McCulloch, D, et al. Quality improvement in chronic illness care: a collaborative approach. Jt Comm J Qual Improv. 2001; 27(2): 63–80. DOI: 10.1016/S1070-3241(01)27007-2 11221012

[7] Frenk, J, Chen, L, Bhutta, ZA, Cohen, J, Crisp, N, Evans, T, et al. Health professionals for a new century: transforming education to strengthen health systems in an interdependent world. The Lancet. 2010; 376(9756): 1923–58. DOI: 10.1016/S0140-6736(10)61854-5 21112623

[8] WHO. (ed.) Modern health care delivery systems, care coordination and the role of hospitals (available at: http://www.euro.who.int/__data/assets/pdf_file/0008/158885/BRU-report-Modern-health-care-delivery-systems.pdf?ua=1). The internal WHO expert meeting on roadmap development, 2012; Copenhagen, Denmark: WHO.

[9] OECD. Improving Value in Health Care: Measuring Quality, 2010; OECD Publishing, Paris.

[10] Iversen, T, Anell, A, Häkkinen, U, Kronborg, C and Ólafsdóttir, T. Coordination of health care in the Nordic countries, 2016; 4(1).

[11] Skudal, KE, Sjetne, IS, Bjertnæs, ØA, Lindahl, AK and Nylenna, M. Commonwealth Fund’s population survey in 11 countries: Norwegian results in 2016 and changes over time, 2016; Norwegian Institute of Public Health. Report No.: 978-82-8082-784-5.

[12] Holmboe, O, Iversen, HH, Sjetne, IS and Skudal, K. Commonwealth Fund’s International Health Policy survey. Results from a comparative population survey in 11 countries, 2011; Report No.: 1890-1298.

[13] The Norwegian Research Council. Evaluation of The Coordination Reform. Proper treatment – at the right place and right time. EVASAM; 2016.

[14] Reeves, S, Lewin, S, Espin, S and Zwarenstein, M. A Conceptual Framework for Interprofessional Teamwork Interprofessional Teamwork for Health and Social Care, 2010; Chapter 4. Copyright © 2010. Blackwell Publishing Ltd DOI: 10.1002/9781444325027.ch4

[15] Szafran, O, Torti, JMI, Kennett, SL and Bell, NR. Family physicians’ perspectives on interprofessional teamwork: Findings from a qualitative study. Journal of interprofessional care, 2018; 32(2): 169–77. DOI: 10.1080/13561820.2017.1395828 29116889

[16] Goldman, J, Meuser, J, Rogers, J, Lawrie, L and Reeves, S. Interprofessional collaboration in family health teams: An Ontario-based study. Can Fam Physician, 2010; 56(10): e368–e74. 20944025 PMC2954101

[17] Xyrichis, A, Reeves, S and Zwarenstein, M. Examining the nature of interprofessional practice: An initial framework validation and creation of the InterProfessional Activity Classification Tool (InterPACT). Journal of interprofessional care, 2017; 1–10. 10.1080/13561820.2017.1408576 29236560

[18] Arksey, H and O’Malley, L. Scoping studies: towards a methodological framework. International Journal of Social Research Methodology, 2005; 8(1): 19–32. DOI: 10.1080/1364557032000119616

[19] Colquhoun, HL, Levac, D, O’Brien, KK, Straus, S, Tricco, AC, Perrier, L, et al. Scoping reviews: time for clarity in definition, methods, and reporting. J Clin Epidemiol, 2014; 67(12): 1291–4. DOI: 10.1016/j.jclinepi.2014.03.013 25034198

[20] Ringard, Å, Sagan, A, Sperre Saunes, I and Lindahl, AK. Norway: Health system review. Health Systems in Transition, 2013; Contract No.: 8. 24434287

[21] Merker, T, Kristiansen, IS and Sæther, EM. Human resources for health care in the Nordic welfare economies: successful today, but sustainable tomorrow? Policy Brief – WHO, 2016; (Pre-publication version).

[22] Lindahl, AK. International Health Care System Profiles: The Norwegian Health Care System: The Commonwealth Fund; 2016 [Available from: http://international.commonwealthfund.org/countries/norway/].

[23] OECD. OECD Reviews of Health Care Quality: Norway 2014. Raising Standards, 2014; OECD Publishing, Paris.

[24] Brelin, P. Presentation at the annual general practice conference Oslo: The Norwegian Association for family medicine [Norsk tittel: Norsk forening for allmennmedisin]; 2017.

[25] Sandelowski, M, Voils, CI, Leeman, J and Crandell, JL. Mapping the Mixed Methods–Mixed Research Synthesis Terrain. Journal of mixed methods research, 2012; 6(4): 317–31. DOI: 10.1177/1558689811427913 23066379 PMC3467952

[26] Hsieh, HF and Shannon, SE. Three approaches to qualitative content analysis. Qual Health Res, 2005; 15(9): 1277–88. DOI: 10.1177/1049732305276687 16204405

[27] Magnussen, S. General practice nurse (GPN) scheme. Evaluation of health care professionals’ satisfaction [Fastlegesykepleier-ordningen: Evaluering av medarbeideres tilfredshet med ordningen. Article in Norwegian]; 2013 Department of Nursing, Faculty of Health, Care and Nursing, Gjøvik, Norway. Contract No.: Report 6.

[28] Godager, G and Iversen, T. Evaluation of the general practice nurse scheme – Improved collaboration between general practitioners and home care nurses in five Norwegian municipalities. [Article in Norwegian. Evaluering av ordningen med fastlegesykepleiere – et tiltak for å bedre samhandlingen mellom fastleger og hjemmesykepleietjenestene i kommunene i Sør-Gudbrandsdalen] Oslo: University of Oslo, Institute of Health and Society; 2013.

[29] Kamps, H and Arnesen, S. GPs collaboration with psychomotoric physiotherapists. [Article in Norwegian. Samhandling mellom allmennlege og psykomotorisk fysioterapeut]. Utposten, 2004; 6.

[30] Kværner, KJ. GPs experiences of communication with nursing homes. [Article in Norwegian. Fastlegers oppfatning av samarbeidet med sykehjem]. Tidsskr Nor Legeforen, 2005; 8(125): 1016–7. 15852075

[31] von Hanno, T. Cancer in primary care in Vestfold in the year of 2000. [Article in Norwegian. Kreftomsorg i allmennpraksis i Vestfold år 2000]. Tidsskr Nor Legeforen, 2000; 29(120): 3557–61. 11188384

[32] Engedal, K, Gausdal, M, Gjora, L and Haugen, PK. Assessment of dementia by a primary health care dementia team cooperating with the family doctor – the Norwegian model. Dement Geriatr Cogn Disord, 2012; 34(5–6): 263–70. DOI: 10.1159/000345435 23183640

[33] Langworthy, JM and Birkelid, J. General practice and chiropractic in Norway: how well do they communicate and what do GPs want to know? J Manipulative Physiol Ther, 2001; 24(9): 576–81. DOI: 10.1067/mmt.2001.118983 11753331

[34] Clancy, A, Gressnes, T and Svensson, T. Public health nursing and interprofessional collaboration in Norwegian municipalities: a questionnaire study. Scand J Caring Sci, 2013; 27(3): 659–68. DOI: 10.1111/j.1471-6712.2012.01079.x 23088191

[35] Kvamme, O and Lothe, MY. Municipal practice consultans improves chronic care [Article in Norwegian: Kommunale praksiskonsulentar gjev betre samhandling for kronisk sjuke]. Utposten, 2014; 4.

[36] Syse, A and Moshina, N. Cancer coordinators’ responsibility of delivering integrated care in Norwegian municipalities. [Article in Norwegian. Kreftkoordinatorers rolle i samhandlingsarbeidet i kreftomsorgen i norske kommuner]. Nordisk Tidsskrift for Helseforskning, 2015; 11(1): 49–65. DOI: 10.7557/14.3476

[37] Graue, M, Dunning, T, Hausken, MF and Rokne, B. Challenges in managing elderly people with diabetes in primary care settings in Norway. Scand J Prim Health Care, 2013; 31(4): 241–7. DOI: 10.3109/02813432.2013.854445 24205973 PMC3860301

[38] Seiger Cronfalk, B, Fjell, A, Carstens, N, Rosseland, LMK, Rongve, A, Ronnevik, DH, et al. Health team for the elderly: a feasibility study for preventive home visits. Prim Health Care Res Dev, 2017; 18(3): 242–52. DOI: 10.1017/S1463423617000019 28215200

[39] Mjell, J and Hjortdahl, P. Improving quality ensurance through local groups in municipal healthcare [Article in Norwegian: Lokale grupper som verktøy for kvalitetssikring i kommunehelsetjenesten]. Tidsskr Nor Legeforen, 2001; 121(14): 1707–9. 11446014

[40] Pedersen, OB and Hovlid, E. Improving quality in general practice through learning networks [Article in Norwegian: Læringsnettverk for bedre kvalitet i allmennpraksis]. Tidsskr Nor Legeforen, 2008; 128(9): 1046–9. 18451884

[41] Mouland, G. Treatment and conntrol of diabetes in general practice. [Article in Norwegian. Diabetes – oppfølging og kontroll i allmennpraksis]. Utposten, 2009; 1.

[42] Bakken, K, Larsen, E, Lindberg, PC, Rygh, E and Hjortdahl, P. Insufficient communication and information regarding patient medication in the primary healthcare [Article in Norwegian: Mangelfull kommunikasjon om legemiddelbruk i primærhelsetjenesten]. Tidsskr Nor Legeforen, 2007; 127(13): 1766–9. 17599123

[43] Bell, HT, Granas, AG, Enmarker, I, Omli, R and Steinsbekk, A. Nurses’ and pharmacists’ learning experiences from participating in interprofessional medication reviews for elderly in primary health care – a qualitative study. BMC Fam Pract. 2017; 18(1): 30 DOI: 10.1186/s12875-017-0598-0 28241789 PMC5330158

[44] Borgen, K, Melby, L, Hellesø, R and Steinsbekk, A. Electronic messaging between home care services and general practitioners [Article in Norwegian: Elektronisk meldingsutveksling mellom hjemmetjenestene og fastleger]. Sykepleien, 2015; 2.

[45] Lyngstad, M, Grimsmo, A, Hofoss, D and Helleso, R. Home care nurses’ experiences with using electronic messaging in their communication with general practitioners. J Clin Nurs, 2014; 23(23–24): 3424–33. DOI: 10.1111/jocn.12590 24646442

[46] Friedman, L and Goes, J. Why integrated health networks have failed. Front Health Serv Manage, 2001; 17(4): 3–28. DOI: 10.1097/01974520-200104000-00002 11436240

[47] Ling, T, Brereton, L, Conklin, A, Newbould, J and Roland, M. Barriers and facilitators to integrating care: experiences from the English Integrated Care Pilots. International journal of integrated care, 2012; 12: e129 DOI: 10.5334/ijic.982 23593044 PMC3601528

[48] WHO. Framework for Action on Interprofessional Education and Collaborative Practice Geneva; 2010 Available at: http://www.who.int/hrh/resources/framework_action/en/. 21174039

[49] Hall, P. Interprofessional teamwork: professional cultures as barriers. Journal of interprofessional care, 2005; 19(Suppl 1): 188–96. DOI: 10.1080/13561820500081745 16096155

[50] Ashton, T. Implementing integrated models of care: the importance of the macro-level context. International journal of integrated care, 2015; 15: e019 DOI: 10.5334/ijic.2247 26528094 PMC4628506

[51] Evans, JM, Grudniewicz, A, Baker, GR and Wodchis, WP. Organizational Capabilities for Integrating Care: A Review of Measurement Tools. Eval Health Prof, 2016; 39(4): 391–420. DOI: 10.1177/0163278716665882 27664122

[52] Leijten, FRM, Struckmann, V, van Ginneken, E, Czypionka, T, Kraus, M, Reiss, M, et al. The SELFIE framework for integrated care for multi-morbidity: Development and description. Health Policy, 2018; 122(1): 12–22. DOI: 10.1016/j.healthpol.2017.06.002 28668222

[53] WHO. Interprofessional Collaborative Practice in Primary Health Care: Nursing and Midwifery Perspectives. Human Resources for Health Observer, 2013; 24.

[54] Leathard, A. Interprofessional Collaboration: From Policy to Practice in Health and Social Care; 2004 Taylor & Francis. 10.1111/j.1475-3588.2005.117_5.x 32806822

[55] Bodolica, V, Spraggon, M and Tofan, G. A structuration framework for bridging the macro–micro divide in health-care governance. Health Expectations: An International Journal of Public Participation in Health Care and Health Policy, 2016; 19(4): 790–804. DOI: 10.1111/hex.12375 26072929 PMC5152727

[56] Bodenheimer, T, Ghorob, A, Willard-Grace, R and Grumbach, K. The 10 Building Blocks of High-Performing Primary Care. Ann Fam Med, 2014; 12(2): 166–71. DOI: 10.1370/afm.1616 24615313 PMC3948764

[57] Lewis, R, Rosen, R, Goodwin, N and Dixon, J. Where next for integrated care organizations in the English NHS? London: The King’s Fund; 2010.

[58] England, N. NHS Leadership Academy England: NHS; 2018 [Available from: https://www.leadershipacademy.nhs.uk/].

[59] Tony, S, Sally, F-D, Susan, N, Brian, ASM and Pam, E. Leadership in interprofessional health and social care teams: a literature review. Leadersh Health Serv. 0(0): null. 10.1108/LHS-06-2016-0026 30234446

[60] Michael, AW, Joanne, L, Regina, E and Jean-Louis, D. Collective leadership for cultures of high quality health care. Journal of Organizational Effectiveness: People and Performance, 2014; 1(3): 240–60. DOI: 10.1108/JOEPP-07-2014-0039

[61] Health SoSf. The Government’s revised mandate to NHS England for 2017–18 In: Health Do, (ed.), 2018; 24 England.

[62] NHS Health Education England. Management England: Health Careers; 2018 Available from: https://www.healthcareers.nhs.uk/explore-roles/management.

[63] Bonciani, M, Barsanti, S and Murante, AM. Is the co-location of GPs in primary care centres associated with a higher patient satisfaction? Evidence from a population survey in Italy. BMC Health Serv Res, 2017; 17: 248 DOI: 10.1186/s12913-017-2187-2 28376886 PMC5379750

[64] Bonciani, M, Schafer, W, Barsanti, S, Heinemann, S and Groenewegen, PP. The benefits of co-location in primary care practices: the perspectives of general practitioners and patients in 34 countries. BMC Health Serv Res, 2018; 18(1): 132 DOI: 10.1186/s12913-018-2913-4 29466980 PMC5822600

[65] Kannampallil, TG, Schauer, GF, Cohen, T and Patel, VL. Considering complexity in healthcare systems. Journal of Biomedical Informatics, 2011; 44(6): 943–7. DOI: 10.1016/j.jbi.2011.06.006 21763459

[66] Lipsitz, LA. Understanding Health Care as a Complex System: The Foundation for Unintended Consequences. JAMA : the journal of the American Medical Association, 2012; 308(3): 243–4. DOI: 10.1001/jama.2012.7551 22797640 PMC3511782

[67] Vachon, B, Désorcy, B, Camirand, M, Rodrigue, J, Quesnel, L, Guimond, C, et al. Engaging primary care practitioners in quality improvement: making explicit the program theory of an interprofessional education intervention. BMC Health Serv Res. 2013; 13: 106 DOI: 10.1186/1472-6963-13-106 23514278 PMC3623830

[68] Weiner, S, Weiner, SJ and Schwartz, A. Listening for What Matters: Avoiding Contextual Errors in Health Care. Oxford University Press; 2016.

[69] West, M, Armit, K, Loewenthal, L, Eckert, R, West, T and Lee, A. Leadership and Leadership Development in Healthcare: The Evidence Base. London: Faculty of Medical Leadership and Management; 2015.

[70] Martin, GP and Waring, J. Leading from the middle: Constrained realities of clinical leadership in healthcare organizations. Health (London, England: 1997), 2013; 17(4): 358–74. DOI: 10.1177/1363459312460704 23060359

[71] NCD-Strategy 2013–2017: For the prevention, diagnosis, treatment and rehabilitation of four noncommunicable diseases: cardiovascular disease, diabetes, COPD and cancer. In: The Norwegian Ministry of Health and Care Services, (ed.); 2012.

[72] Entwistle, T, Bristow, G, Hines, F, Donaldson, S and Martin, S. The Dysfunctions of Markets, Hierarchies and Networks in the Meta-governance of Partnership. Urban Studies, 2007; 44(1): 63–79. DOI: 10.1080/00420980601023836

[73] Willem, A and Gemmel, P. Do governance choices matter in health care networks?: an exploratory configuration study of health care networks. BMC Health Serv Res, 2013; 13(1): 229 DOI: 10.1186/1472-6963-13-229 23800334 PMC3727985

[74] Kodner, DL. All together now: a conceptual exploration of integrated care. Healthcare quarterly (Toronto, Ont), 2009; 13(Spec No: 6–15). 10.12927/hcq.2009.21091 20057243

[75] Feinberg, LF. Moving Toward Person- and Family-Centered Care. Public Policy & Aging Report, 2014; 24(3): 97–101. DOI: 10.1093/ppar/pru027

[76] Green, LW, Ottoson, JM, García, C and Hiatt, RA. Diffusion Theory and Knowledge Dissemination, Utilization, and Integration in Public Health. Annu Rev Public Health, 2009; 30(1): 151–74. DOI: 10.1146/annurev.publhealth.031308.100049 19705558

[77] May, CR, Mair, F, Finch, T, MacFarlane, A, Dowrick, C, Treweek, S, et al. Development of a theory of implementation and integration: Normalization Process Theory. Implementation Science, 2009; 4(1): 29 DOI: 10.1186/1748-5908-4-29 19460163 PMC2693517

[78] Bodolica, V and Spraggon, M. Clinical Governance Infrastructures and Relational Mechanisms of Control in Healthcare Organizations. Journal of Health Management, 2014; 16(2): 183–98. DOI: 10.1177/0972063414526126

[79] Putera, I. Redefining Health: Implication for Value-Based Healthcare Reform. Cureus, 2017; 9(3): e1067. 38348426 10.7759/cureus.1047 PMC10860730

[80] Services TNMoHaC. Meld. St. 16 (2010–2011). National Health and Care Services Plan (2011–2015). Report to the Storting (white paper); 2012.

[81] The Ministry of Health and Care Services. Meld. St. 26 (2014–2015). The primary health and care services of tomorrow – localised and integrated; 2015.

[82] The Norwegian Ministry of Health and Care Services. The Coordination Reform: Proper treatment – at the right place and right time. Report No. 47 (2008–2009) to the Storting; 2008–2009.

[83] Healthcare Act [Article in Norwegian: Helse- og omsorgstjenesteloven 2012]. In: The Norwegian Ministry of Health and care services, (ed.); 2012.

[84] Public health Act [Article in Norwegian: Lov om folkehelsearbeid (folkehelseloven)]; 2011.

[85] Reeves, S, Macmillan, K and Van Soeren, M. Leadership of interprofessional health and social care teams: a socio-historical analysis. J Nurs Manag. 2010; 18(3): 258–64. DOI: 10.1111/j.1365-2834.2010.01077.x 20546465

[86] D’Amour, D, Goulet, L, Labadie, JF, Martin-Rodriguez, LS and Pineault, R. A model and typology of collaboration between professionals in healthcare organizations. BMC Health Serv Res, 2008; 8: 188 DOI: 10.1186/1472-6963-8-188 18803881 PMC2563002

[87] Collaborative CIH. A National Interprofessional Competency Framework. Vancouver, Canada; 2010.

[88] Mulvale, G, Embrett, M and Razavi, SD. ‘Gearing Up’ to improve interprofessional collaboration in primary care: a systematic review and conceptual framework. BMC Fam Pract, 2016; 17: 83 DOI: 10.1186/s12875-016-0492-1 27440181 PMC4955241

[89] Evans, JM, Grudniewicz, A, Baker, GR and Wodchis, WP. Organizational Context and Capabilities for Integrating Care: A Framework for Improvement. International journal of integrated care, 2016; 16(3): 15 DOI: 10.5334/ijic.2416 PMC5388061 28413366

[90] Steele Gray, C, Wodchis, W, Baker, G, Carswell, P, Kenealy, T, McKillop, A, et al. Mapping for Conceptual Clarity: Exploring Implementation of Integrated Community-Based Primary Health Care from a Whole Systems Perspective; 2018. 10.5334/ijic.3082 PMC6095076 30127683

[91] Santana, MJ, Manalili, K, Jolley, RJ, Zelinsky, S, Quan, H and Lu, M. How to practice person-centred care: A conceptual framework. Health Expect, 2018; 21(2): 429–40. DOI: 10.1111/hex.12640 29151269 PMC5867327

[92] Chung, KSK. Understanding Decision Making through Complexity in Professional Networks. Advances in Decision Sciences, 2014; 2014: 10 DOI: 10.1155/2014/215218

[93] Reeves, S, Pelone, F, Harrison, R, Goldman, J and Zwarenstein, M. Interprofessional collaboration to improve professional practice and healthcare outcomes. The Cochrane database of systematic reviews, 2017; 6 DOI: 10.1002/14651858.CD000072.pub3 PMC6481564 28639262

[94] MacNaughton, K, Chreim, S and Bourgeault, IL. Role construction and boundaries in interprofessional primary health care teams: a qualitative study. BMC Health Serv Res, 2013; 13: 486 DOI: 10.1186/1472-6963-13-486 24267663 PMC4222600

[95] Scott, TIM, Mannion, R, Davies, HTO and Marshall, MN. Implementing culture change in health care: theory and practice. Int J Qual Health Care, 2003; 15(2): 111–8. DOI: 10.1093/intqhc/mzg021 12705704

[96] Huw, CJ, Joanna, L and Carl, M. Transforming general practice: the redistribution of medical work in primary care. Sociol Health Illn, 2003; 25(1): 71–92. DOI: 10.1111/1467-9566.t01-1-00325 14498945

[97] Clancy, TR, Effken, JA and Pesut, D. Applications of complex systems theory in nursing education, research, and practice. Nurs Outlook, 2008; 56(5): 248–56.e3. DOI: 10.1016/j.outlook.2008.06.010 18922279

[98] Bem, C. Social governance: a necessary third pillar of healthcare governance. J R Soc Med, 2010; 103(12): 475–7. DOI: 10.1258/jrsm.2010.100315 21127325 PMC2996525

[99] Reeves, S, Lewin, S, Espin, S and Zwarenstein, M. Interprofessional Teamwork for Health and Social Care. Blackwell Publishing Ltd; 2010 DOI: 10.1002/9781444325027

