# Supplementary material for: A Scoping Review of Facilitators of Multi-Professional Collaboration in Primary Care
Source: Int J Integr Care. 2018 Aug 30;18(3):13. doi: 10.5334/ijic.3959 (PMC6137624; doi:10.5334/ijic.3959)
Supplement: Appendix 1 — Search strategy. [file ijic-18-3-3959-s1.pdf]

Appendix 1. Search strategy

Contact: Monica Sørensen
Librarian: Marita Heintz

Duoblet control EndNote Before control: 1011
After control: 764

Database: Ovid MEDLINE(R) Epub Ahead of Print, In-Process & Other Non-Indexed Citations, Ovid MEDLINE(R) Daily and Ovid MEDLINE(R) <1946 to Present>
Date: July 12th 2017
Number of hits: 285 (267 internal doublets discarded)

1	exp General Practice/ or general practitioners/ or physicians, family/ or physicians, primary care/ or Primary Health Care/	148688
2	(General practice* or Family Practice* or general medical practice* or general practitioner* or family doctor* or family physician* or family practitioner* or general physician* or family medicine or primary care or primary health care or primary healthcare or first line care or primary medical care).tw.	197241
3	1 or 2	247345
4	medical secretaries/ or medical receptionists/ or exp nurses/ or nursing staff/ or nutritionists/ or pharmacists/ or physical therapists/ or laboratory personnel/ or medical laboratory personnel/ or social workers/	118962
5	(medical secretar* or medical receptionist* or nurse* or (nursing adj1 (staff or personnel)) or nutritionist* or Dietician* or Dietitian* or pharmacist* or physical therapist* or Physiotherapist or (laboratory adj1 (personnel or staff or worker* or Technician* or Scientist or Assistant)) or social worker*).tw.	298169
6	Patient care team/ or Intersectoral Collaboration/	60911
7	((Multiprofessional or interprofessional or multidisciplin* or interdisciplin* or Intersectoral) adj3 (care or healthcare or practice or collaboration? or coordination or "co ordination" or program*)).tw.	16631
8	(team* or skillmix or "skill mix" or "Collaborative care").tw.	132939
9	or/4-8	509561
10	3 and 9	34097
11	limit 10 to yr="2000 -Current"	24077
12	exp Norway/	34757
13	(norway or Norwegian? or norge).tw,cp,in,lg.	167006

14	(sykehus* or sjukehus* or ((universitet* or University or univ) and (haukeland or nordnorge or norge* or bergen or stavanger or tromso or tromsoe or trondheim or levanger or gjovik or gjoevik or harstad or lillehammer or narvik or nesna or stord or haugesund or volda or aalesund or alesund)) or sentralsjukehus* or sentralsykehus* or Finnmarkssykehuset or Helgelandssykehuset or Nordlandssykehuset or innlandet or "Olav? Hospital?" or revmatismesykehus or lungesykehus or "Hospitalet Betanien" or Kysthospitalet or Aleris or Feiringklinikken or Glittreklinikken or "Hjertesenteret i Oslo" or "Medi 3" or "Volvat Medisinske Senter" or "Helse Vest" or "Helse Stavanger" or "Helse fonna" or "helse bergen" or "helse forde" or "helse foerde" or sjukehusapotek* or sykehusapotek* or "helse midt norge" or "helse midtnorge" or "Ambulanse Midtnorge" or "Ambulanse Midt norge" or "helse nord" or "Helse Sorost" or "Helse Sor ost" or "Helse Soeroest" or "Helse Soer oest" or sunnaas or sunnas or sorlandet or soerlandet or "Vestre Viken").cp,in,tw.	45664
15	(Akershus or Aust-Agder or Agder or Buskerud or Finnmark or Hedmark or Hordaland or Romsdal or Nordland or Nordtrondelag or Trondelag or Nordtroendelag or Troendelag or Oppland or Oslo or Rogaland or Fjordane or Sortrondelag or Soertroendelag or Telemark or Troms or Vestagder or Vestfold or Ostfold or Oestfold).cp,in,tw.	61502
16	tidsskrift for den norske laegeforening.jn.	31434
17	or/12-16	173303
18	11 and 17	285

Database: Embase <1974 to 2017 July 11>
Date: Juli 12th 2017
Number of hits: 363 (349 internal doublets discarded)

1	general practice/ or general practitioner/ or family medicine/ or exp primary health care/	264338
2	(General practice* or Family Practice* or general medical practice* or general practitioner* or family doctor* or family physician* or family practitioner* or general physician* or GP or GPS or family medicine or primary care or primary health care or primary healthcare or first line care or primary medical care).tw.	286439
3	1 or 2	376071
4	exp nurse/ or medical secretary/ or medical receptionist/ or nursing staff/ or dietitian/ or pharmacist/ or physiotherapist/ or laboratory personnel/ or clinical laboratory personnel/ or social worker/	288619
5	(medical secretar* or medical receptionist* or nurse* or (nursing adj1 (staff or personnel)) or nutritionist* or Dietician* or Dietitian* or pharmacist* or physical therapist* or Physiotherapist or (laboratory adj1 (personnel or staff or worker* or Technician* or Scientist* or Assistant*)) or social worker*).tw.	367243

6	intersectoral collaboration/ or skill mix/	544
7	((Multiprofessional or interprofessional or multidisciplin* or interdisciplin* or Intersectoral) adj3 (care or healthcare or practice or collaboration? or coordination or "co ordination" or program*)).tw.	23225
8	(team* or skillmix or "skill mix" or "Collaborative care").tw.	193154
9	or/4-8	651421
10	3 and 9	49438
11	limit 10 to yr="2000 -Current"	39349
12	norway/ or "svalbard and jan mayen"/	37646
13	(norway or Norwegian? or norge).cp,in,ad,tw,lg.	264296
14	(sykehus* or sjukehus* or ((universitet* or University or univ) and (haukeland or nordnorge or norge* or bergen or stavanger or tromso or tromsoe or trondheim or levanger or gjovik or gjoevik or harstad or lillehammer or narvik or nesna or stord or haugesund or volda or aalesund or alesund)) or sentralsjukehus* or sentralsykehus* or Finnmarkssykehuset or Helgelandssykehuset or Nordlandssykehuset or innlandet or "Olav? Hospital?" or revmatismesykehus or lungesykehus or "Hospitalet Betanien" or Kysthospitalet or Aleris or Feiringklinikken or Glittreklinikken or "Hjertesenteret i Oslo" or "Medi 3" or "Volvat Medisinske Senter" or "Helse Vest" or "Helse Stavanger" or "Helse fonna" or "helse bergen" or "helse forde" or "helse foerde" or sjukehusapotek* or sykehusapotek* or "helse midt norge" or "helse midtnorge" or "Ambulanse Midtnorge" or "Ambulanse Midt norge" or "helse nord" or "Helse Sorost" or "Helse Sor ost" or "Helse Soeroest" or "Helse Soer oest" or sunnaas or sunnas or sorlandet or soerlandet or "Vestre Viken").cp,in,ad,ti,ab.	77249
15	(Akershus or Aust-Agder or Agder or Buskerud or Finnmark or Hedmark or Hordaland or Romsdal or Nordland or Nordtrondelag or Trondelag or Nordtroendelag or Troendelag or Oppland or Oslo or Rogaland or Fjordane or Sortrondelag or Soertroendelag or Telemark or Troms or Vestagder or Vestfold or Ostfold or Oestfold).cp,in,ad,ti,ab.	104246
16	(oslonorway or bergennorway or sandnesnorway or stavangernorway or trondheimnorway or tromsonorway or tromsoenorway or Akershusnorway or Austagdernorway or Agdernorway or Buskerudnorway or Finnmarknorway or Hedmarknorway or Hordalandnorway or Romsdalnorway or Nordlandnorway or Nordtrondelagnorway or Nordtroendelagnorway or Trondelagnorway or Troendelagnorway or Opplandnorway or Rogalandnorway or Fjordanenorway or Sortrondelagnorway or Sortroendelagnorway or Telemarknorway or Tromsnorway or Vestagdernorway or Vestfoldnorway or Ostfoldnorway or Oestfoldnorway).cp,in,ad,ti,ab.	391
17	(tidsskrift for den norske laegeforening or tidsskrift for den norske laegeforening tidsskrift for praktisk or tidsskrift for den norske laegeforening tidsskrift for praktisk medicin ny raekke).jn.	30584

18	or/12-17	274485
19	11 and 18	482
20	Elsevier.cr.	22446681
21	19 and 20	363

Database: PsycINFO 1806 to July Week 1 2017
Date: July 12. 2017
Number of hits: 86

1	family physicians/ or family medicine/ or general practitioners/ or primary health care/	22345
2	(General practice* or Family Practice* or general medical practice* or general practitioner* or family doctor* or family physician* or family practitioner* or general physician* or GP or GPS or family medicine or primary care or primary health care or primary healthcare or first line care or primary medical care).tw.	45940
3	1 or 2	48475
4	exp nurses/ or exp social workers/ or pharmacists/ or physical therapists/	39667
5	(medical secretar* or medical receptionist* or nurse* or (nursing adj1 (staff or personnel)) or nutritionist* or Dietician* or Dietitian* or pharmacist* or physical therapist* or Physiotherapist or (laboratory adj1 (personnel or staff or worker* or Technician* or Scientist* or Assistant*)) or social worker*).tw.	84588
6	Interdisciplinary Treatment Approach/	6644
7	((Multiprofessional or interprofessional or multidisciplin* or interdisciplin* or Intersectoral) adj3 (care or healthcare or practice or collaboration? or coordination or "co ordination" or program*)).tw.	6179
8	(team* or skillmix or "skill mix" or "Collaborative care").tw.	66500
9	or/4-8	155441
10	3 and 9	8172
11	limit 10 to yr="2000 -Current"	6961
12	(norway or Norwegian? or norge).in,cq,lo,tw,lg,ca.	32286
13	(sykehus* or sjukehus* or ((universitet* or University or univ) and (haukeland or nordnorge or norge* or bergen or stavanger or tromso or tromsoe or trondheim or levanger or gjovik or gjoevik or harstad or lillehammer or narvik or nesna or stord or haugesund or volda or aalesund or alesund)) or sentralsjukehus* or sentralsykehus* or Finnmarkssykehuset or Helgelandssykehuset or Nordlandssykehuset or innlandet or "Olav? Hospital?" or revmatismesykehus or lungesykehus or	10367

	"Hospitalet Betanien" or Kysthospitalet or Aleris or Feiringklinikken or Glittreklinikken or "Hjertesenteret i Oslo" or "Medi 3" or "Volvat Medisinske Senter" or "Helse Vest" or "Helse Stavanger" or "Helse fonna" or "helse bergen" or "helse forde" or "helse foerde" or sjukehusapotek* or sykehusapotek* or "helse midt norge" or "helse midtnorge" or "Ambulanse Midtnorge" or "Ambulanse Midt norge" or "helse nord" or "Helse Sorost" or "Helse Sor ost" or "Helse Soeroest" or "Helse Soer oest" or sunnaas or sunnas or sorlandet or soerlandet or "Vestre Viken").cq,in,tw,ca.	
14	(Akershus or Aust-Agder or Agder or Buskerud or Finnmark or Hedmark or Hordaland or Romsdal or Nordland or Nordtrondelag or Trondelag or Nordtroendelag or Troendelag or Oppland or Oslo or Rogaland or Fjordane or Sortrondelag or Soertroendelag or Telemark or Troms or Vestagder or Vestfold or Ostfold or Oestfold).cq,in,tw,ca.	15314
15	(tidsskrift for den norske laegeforening or tidsskrift for norsk psykologforening).jn.	1416
16	or/12-15	32950
17	11 and 16	86

Database: Cinahl
Date: July 12th 2017
Number of hits:30

S15	S11 AND S14	30
S14	S12 OR S13	8,754
S13	TI ((norway or Norwegian# or norge)) OR AB ((norway or Norwegian# or norge))	5,315
S12	(MH "Norway")	7,229
S11	S3 AND S9 Limiters - Published Date: 20000101-20171231	6,301
S10	S3 AND S9	7,479
S9	S4 OR S5 OR S6 OR S7 OR S8	431,292
S8	TI ((team* or skillmix or "skill mix" or "Collaborative care")) OR AB ((team* or skillmix or "skill mix" or "Collaborative care"))	55,011
S7	TI (((Multiprofessional or interprofessional or multidisciplin* or interdisciplin* or Intersectoral) N2 (care or healthcare or practice or collaboration# or coordination or "co ordination" or program*))) OR AB (((Multiprofessional or interprofessional or multidisciplin* or interdisciplin* or Intersectoral) N2 (care or healthcare or practice or collaboration# or coordination or "co ordination" or program*)))	7,494
S6	(MH "Multidisciplinary Care Team+") OR (MH "Skill Mix+")	28,814
S5	TI ((medical secretar* or medical receptionist* or nurse* or (nursing N0 (staff or personnel)) or nutritionist* or Dietician* or Dietitian* or pharmacist* or physical therapist* or Physiotherapist or (laboratory N0 (personnel or staff or worker* or	270,430

	Technician* or Scientist or Assistant)) or social worker*)) OR AB ((medical secretar* or medical receptionist* or nurse* or (nursing N0 (staff or personnel)) or nutritionist* or Dietician* or Dietitian* or pharmacist* or physical therapist* or Physiotherapist or (laboratory N0 (personnel or staff or worker* or Technician* or Scientist or Assistant)) or social worker*))	
S4	(MH "Nurses+") OR (MH "Dietitians") OR (MH "Physical Therapists") OR (MH "Pharmacists") OR (MH "Laboratory Personnel+") OR (MH "Social Workers")	192,685
S3	S1 AND S2	29,399
S2	TI (("General practice*" or "Family Practice*" or "general medical practice*" or "general practitioner*" or "family doctor*" or "family physician*" or "family practitioner*" or "general physician*" or GP or GPS or "family medicine" or "primary care" or "primary health care" or "primary healthcare" or "first line care" or "primary medical care")) OR AB ((("General practice*" or "Family Practice*" or "general medical practice*" or "general practitioner*" or "family doctor*" or "family physician*" or "family practitioner*" or "general physician*" or GP or GPS or "family medicine" or "primary care" or "primary health care" or "primary healthcare" or "first line care" or "primary medical care"))	61,075
S1	(MH "Family Practice") OR (MH "Physicians, Family") OR (MH "Primary Health Care")	51,921

Database: Cochrane Database of Systematic Reviews : Issue 7 of 12, July 2017, Database of Abstracts of Reviews of Effect : Issue 2 of 4, April 2015, Cochrane Central Register of Controlled Trials : Issue 6 of 12, June 2017, NHS Economic Evaluation Database : Issue 2 of 4, April 2015, Health Technology Assessment Database : Issue 4 of 4, October 2016
Date: July 12th 2017
Number of hits: 36 (Other: 4, Trials: 27, Eco: 5)

ID	Search	Hits
#1	[mh "General Practice"]	2553
#2	[mh ^"general practitioners"]	182
#3	[mh ^"physicians, family"]	481
#4	[mh ^"physicians, primary care"]	127
#5	[mh ^"Primary Health Care"]	4032
#6	("General practice" or "Family Practice" or "general medical practice" or "general practitioner*" or "family doctor*" or "family physician*" or "family practitioner*" or "general physician*" or GP or GPS or "family medicine" or "primary care" or "primary health care" or "primary healthcare" or "first line care" or "primary medical care"):ti,ab,kw	21038
#7	("General practice" or "Family Practice" or "general medical practice" or "general practitioner*" or "family doctor*" or "family physician*" or "family practitioner*" or "general physician*" or GP or GPS or "family medicine" or "primary care" or "primary health care" or "primary healthcare" or "first line care" or "primary medical care”) in Other Reviews, Technology Assessments and Economic Evaluations	4625
#8	{or #1-#7}	24430

#9	[mh ^"Patient care team"]	1691
#10	[mh ^"Intersectoral Collaboration"]	7
#11	((Multiprofessional or interprofessional or multidisciplin* or interdisciplin* or Intersectoral) near/3 (care or healthcare or practice or collaboration* or coordination or "co ordination" or program*)):ti,ab,kw	1330
#12	((Multiprofessional or interprofessional or multidisciplin* or interdisciplin* or Intersectoral) near/3 (care or healthcare or practice or collaboration* or coordination or "co ordination" or program*)) in Other Reviews, Technology Assessments and Economic Evaluations	183
#13	(team* or skillmix or "skill mix" or "Collaborative care"):ti,ab,kw	9104
#14	(team* or skillmix or "skill mix" or "Collaborative care") in Other Reviews, Technology Assessments and Economic Evaluations	1429
#15	[mh ^"medical secretaries"]	2
#16	[mh ^"medical receptionists"]	2
#17	[mh nurses]	1167
#18	[mh ^"nursing staff"]	199
#19	[mh ^nutritionists]	19
#20	[mh ^pharmacists]	573
#21	[mh ^"physical therapists"]	70
#22	[mh ^"laboratory personnel"]	7
#23	[mh ^"medical laboratory personnel"]	11
#24	[mh ^"social workers"]	2
#25	("medical secretar*" or "medical receptionist*" or nurse* or (nursing near/1 (staff or personnel)) or nutritionist* or Dietician* or Dietitian* or pharmacist* or "physical therapist*" or Physiotherapist or (laboratory near/1 (personnel or staff or worker* or Technician* or Scientist* or Assistant*)) or "social worker*"):ti,ab,kw	19798
#26	("medical secretar*" or "medical receptionist*" or nurse* or (nursing near/1 (staff or personnel)) or nutritionist* or Dietician* or Dietitian* or pharmacist* or "physical therapist*" or Physiotherapist or (laboratory near/1 (personnel or staff or worker* or Technician* or Scientist* or Assistant*)) or "social worker*") in Other Reviews, Technology Assessments and Economic Evaluations	2735
#27	{or #9-#26}	30566
#28	[mh norway]	856
#29	(norway or Norwegian* or Norge):ti,ab,kw	2316
#30	(norway or Norwegian* or Norge) in Other Reviews, Technology Assessments and Economic Evaluations	360
#31	{or #28-#30}	2598
#32	#8 and #27 and #31	38
#33	#8 and #27 and #31 Publication Year from 2000 to 2017	36

Database: Epistemonikos
Date: July 12 th 2017
Number of hits:12

("General practice" or "Family Practice" or "general medical practice" or "general practitioner" or "general practitioners" or "family doctor" or "family doctors" or "family physician*" or "family practitioner" or "family practitioners" or "general physician" or "general physicians" or GP or GPS or "family medicine" or "primary care" or "primary health care" or "primary healthcare" or "first line care" or "primary medical care") AND (Multiprofessional or interprofessional or multidisciplin* or interdisciplin* or Intersectoral or team* or skillmix or "skill mix" or "Collaborative care" or "medical secretaries" or "medical secretary" or "medical receptionist" or "medical receptionists" or nurse* or "nursing staff" or "nursing personnel" or nutritionist* or Dietician* or Dietitian* or pharmacist* or "physical therapist" or "physical therapists" or "laboratory personnel" or "laboratory staff" or "laboratory worker" or "laboratory workers" or "laboratory technician" or "laboratory technicians" or "laboratory scientist" or "laboratory scientist" or "social worker" or "social workers") AND (norway or norwegian* or norge)
-avgrenset 2000-2017

Database: SveMed+
Date: July 13th 2017
Number of hits: 202
Comment: No time limit

1	exp:"General Practice"	3206
2	noexp:"general practitioners"	243
3	noexp:"physicians, family"	1292
4	noexp:"physicians, primary care"	8
5	noexp:"Primary Health Care"	2036
6	"General practice"	3368
7	"Family Practice"	2678
8	"general medical practice"	1
9	"general practitioner"	807
10	"family doctor"	25
11	"family physician"	1304
12	"family practitioner"	130
13	"general physician"	2
14	"family medicine"	9
15	"primary care"	3808
16	"primary health care"	4490
17	"primary healthcare"	3744
18	"first line care"	0
19	"primary medical care"	4
20	#1 OR #2 OR #3 OR #4 OR #5 OR #6 OR #7 OR #8 OR #9 OR #10 OR #11 OR #12 OR #13 OR #14 OR #15 OR #16 OR #17 OR #18 OR #19	7694
21	noexp:"medical secretaries"	25

22	noexp:"medical receptionists"	3
23	exp:"nurses"	2042
24	noexp:"nursing staff"	589
25	noexp:"nutritionists"	15
26	noexp:"pharmacists"	168
27	noexp:"physical therapists"	63
28	noexp:"laboratory personnel"	3
29	noexp:"medical laboratory personnel"	53
30	noexp:"social workers"	0
31	"medical secretary"	28
32	"medical receptionist"	3
34	"nursing staff"	932
35	"nursing personnel"	2048
36	"nutritionist"	18
37	"Dietician"	20
38	"Dietitian"	28
39	"pharmacist"	182
40	"physical therapist"	159
41	"Physiotherapist"	218
42	"laboratory personnel"	56
43	"laboratory staff"	0
44	"laboratory worker"	0
45	"laboratory technician"	57
46	"laboratory scientist"	57
47	"social worker"	34
48	sykepleier	4383
49	sykepleiere	2454
50	#21 OR #22 OR #23 OR #24 OR #25 OR #26 OR #27 OR #28 OR #29 OR #30 OR #31 OR #32 OR #34 OR #35 OR #36 OR #37 OR #38 OR #39 OR #40 OR #41 OR #42 OR #43 OR #44 OR #45 OR #46 OR #47 OR #48 OR #49	7182
51	#20 AND #50	641
52	lang:"Nor"	31152
53	#51 AND #52	183
54	exp:"Norway"	12435
55	Norway	12490
56	norwegian	1362
57	norge	12491
58	norsk	20403
59	nordmenn	14
60	Nordmænd	0
61	Norrmän	0

62	#54 OR #55 OR #56 OR #57 OR #58 OR #59 OR #60 OR #61	24581
63	#51 AND #62	95
64	#53 OR #63	202

Database: NorArt
Date: July 13th 2017
Number of hits: 29
Comment: No time limit

(Sykepleier* OR fysioterapeut* OR ernæringsfysiolog* OR farmasøyt* OR legesekretær* OR laboratorietekniker OR laborant OR sosionom OR sosialarbeider) AND (legekontor* OR fastlegekontor* OR primærhelse*)
