# Supplementary material for: A Scoping Review of Facilitators of Multi-Professional Collaboration in Primary Care
Source: Int J Integr Care. 2018 Aug 30;18(3):13. doi: 10.5334/ijic.3959 (PMC6137624; doi:10.5334/ijic.3959)
Supplement: Appendix 2 — Data extraction form. [file ijic-18-3-3959-s2.pdf]

Appendix 2. Data extraction form

Reference	
	First author
	Title
	Journal reference
Inclusion Criteria	
	English or Scandinavian language article publish after year 2000
Qualitative or quantitative study that comply with all of the following criteria:	
	a) One or more GPs involved in multiprofessional collaboration within general practice, community care and/or primary health care in Norway
	b) The purpose of the intervention is to improve quality or safety of multiprofessional collaboration, to improve multiprofessional communication or the aim of the study is to describe multiprofessional collaboration
Description of Study	
	Purpose/aim of study
	Design
	Method for inclusion of participants
	Description of the qualitative or quantitative methods of data analyses
	Description of instruments and procedures used to assess effectiveness or contribution of collaboration/intervention
	Description of context factors relevant for study experiences and outcomes
	(If relevant: Description of methods used to assure data quality and training of participants)
Study Population and team composition	
	Description of teams and responsibilities of members of the team
	Intervention type: Description of the initiative and information about the collaboration, team roles, and modes of communication
	(If relevant: Change in practice compared to practice as usual)
	Description of health professions, work place and clinical setting, geographical location
	(If relevant: Description of care recipient group, diagnoses or other characteristics, setting of initiative (home based care, general practice, nursing home), geographical location
	Number of participants
Qualitative Study (any study design)	
	Experiences of being part of team
	Health care professionals perceived facilitators, barriers and benefits of multiprofessional collaboration
	Description of qualitative design
Description of collaboration or intervention	
	Any of the following if relevant:
	Assessments
	Medication use
	Flow of Information
	Quality improvement
	Leadership, organizational culture
	User experiences
	Patient satisfaction
Outcomes (Results)	
	Impact of collaboration or initiative/intervention on any outcome
	Objective measurement of impact or effectiveness of the collaboration or intervention
	Lessons learned of factors that could affect benefits, facilitators, harms, barriers, or failures of the intervention
	Implications of study results
Evaluation	
Limitations	
Strengths	
Implications of study results	
(If relevant: Economic aspects)	
General reflexions and gaps in research	

As scoping studies does not seek to evaluate the quality of evidence, the relative weight of evidence in favour of any particular intervention is not addressed (Arksey and O'Malley).
