# Supplementary material for: A Scoping Review of Facilitators of Multi-Professional Collaboration in Primary Care
Source: Int J Integr Care. 2018 Aug 30;18(3):13. doi: 10.5334/ijic.3959 (PMC6137624; doi:10.5334/ijic.3959)
Supplement: Appendix 3 — List of consulted stakeholders. [file ijic-18-3-3959-s3.pdf]

Appendix 3. **List of consulted stakeholders**

Name	Title	Affilation
Bjørn Gjelsvik	MD, PhD	University of Oslo, Institute for Health and Society, Department of General Practice
Unni-Berit Skjervheim	MD, District medical officer	Nes Municipality, Health and Social Services
Rolf Windspol	Senior Advisor, Head of the national primary care team program	The Norwegian Directorate of Health
Helene Aksøy	Senior advisor, RN MNSc	Norwegian Nurses Organization, Department of Nursing and Health Politics
